# Probing Glycosaminoglycan–Protein Interactions: Applications of Surface Plasmon Resonance

**DOI:** 10.3390/bios16020071

**Published:** 2026-01-25

**Authors:** Changkai Bu, Lin Pan, Lianli Chi, Vitor H. Pomin, Jonathan S. Dordick, Chunyu Wang, Fuming Zhang

**Affiliations:** 1 National Glycoengineering Research Center, Shandong University, Qingdao 266237, China; buc@rpi.edu (C.B.); lianlichi@sdu.edu.cn (L.C.); 2 Center for Biotechnology and Interdisciplinary Studies, Rensselaer Polytechnic Institute, Troy, NY 12180, USA; panl5@rpi.edu (L.P.); dordick@rpi.edu (J.S.D.); 3 Department of BioMolecular Sciences, Research Institute of Pharmaceutical Sciences, School of Pharmacy, The University of Mississippi, Oxford, MS 38677, USA; vpomin@olemiss.edu

**Keywords:** glycosaminoglycan, surface plasmon resonance, biosensor, protein interactions, heparin, binding kinetics, extracellular matrix, glycomics

## Abstract

Glycosaminoglycans (GAGs) are highly negatively charged polysaccharides that play essential roles in numerous physiological and pathological processes through their interactions with proteins. These interactions govern cellular signaling, inflammation, coagulation, and recognition. Surface Plasmon Resonance (SPR) has emerged as a key biophysical technique for label-free, real-time characterization of biomolecular interactions, offering insights into binding kinetics, affinity, and specificity. SPR-based approaches to glycosaminoglycan–protein interaction studies offer powerful tools for elucidating the roles of GAGs in a wide range of physiological and pathological processes. In this review, we systematically discuss experimental strategies, data analysis methods, and representative applications of SPR-based glycosaminoglycan–protein interactions. Special attention is given to the challenges associated with GAG heterogeneity and immobilization, as well as recent technological advances that enhance sensitivity and throughput. To our knowledge, this review represents one of the first systematic and up-to-date summaries specifically focused on recent advances in applying SPR to the study of glycosaminoglycan–protein interactions.

## 1. Introduction

**Figure 1:**
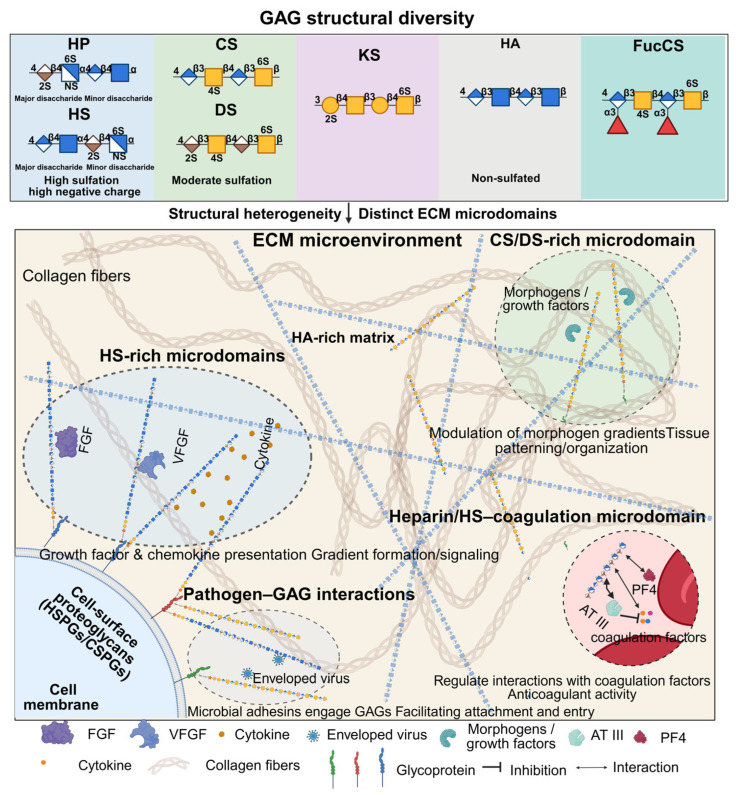
Structural diversity of GAGs and their roles within the extracellular matrix (ECM) microenvironment.

Glycosaminoglycans (GAGs) are a diverse family of linear, highly negatively charged polysaccharides that play critical roles in cellular communication, tissue development, and homeostasis. These molecules, including heparin, heparan sulfate, chondroitin sulfate, dermatan sulfate, keratan sulfate, and hyaluronan, are widely distributed on cell surfaces and within the extracellular matrix, where they interact with a broad range of proteins including growth factors, chemokines, coagulation factors, and microbial adhesins [1,2,3]. Through such interactions, GAGs influence processes such as angiogenesis, inflammation, wound repair, and pathogen entry (Figure 1) [4,5,6]. Another unique GAG, both structurally and functionally diverse and widely used in research, is fucosylated chondroitin sulfate (FucCS), which is found exclusively in sea cucumbers [7]. Recently, Vallet et al. (2022) reported GAG Interactome 2.0, comprising 3464 GAG-binding proteins and 4290 GAG–protein interactions [8]. The biological activities of GAGs are highly dependent on their structural heterogeneity, particularly the degree and pattern of sulfation and the length of the polysaccharide chains [9]. These variations endow GAGs with the ability to engage in highly specific and often multivalent interactions with proteins, despite the overall electrostatic nature of their binding. The complexity of GAG–protein interactions poses significant analytical challenges, especially in determining binding affinity, kinetics, and specificity under physiologically relevant conditions.

**Figure 2:**
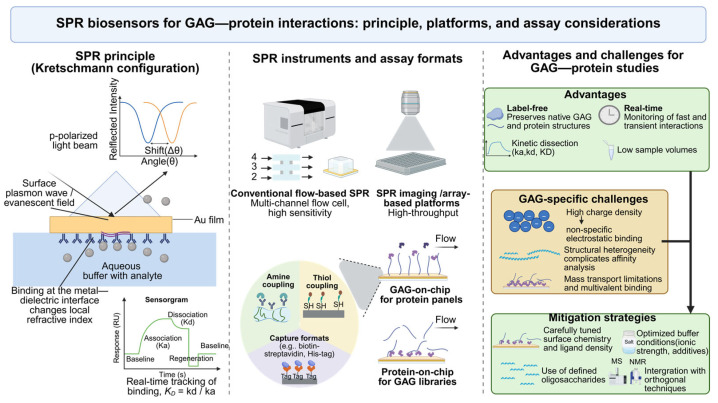
SPR biosensors for dissecting GAG–protein interactions.

Surface Plasmon Resonance (SPR) is a powerful, label-free biosensing technology that allows real-time monitoring of biomolecular interactions (Figure 2). It is particularly well-suited for studying GAG–protein interactions due to its sensitivity, quantitative output, and ability to resolve kinetic parameters such as association (*ka*) and dissociation (*kd*) rate constants, as well as the equilibrium dissociation constant (*K_D_*) [10,11]. SPR has been successfully applied to a variety of systems involving GAGs, including the characterization of heparin-binding growth factors, analysis of anticoagulant mechanisms, and screening of GAG-based inhibitors [12,13,14].

Despite its advantages, SPR-based analysis of GAG–protein interactions presents specific technical challenges, including the immobilization of highly charged and flexible GAG chains, potential non-specific binding, and the interpretation of multivalent or cooperative binding modes. Addressing these challenges has driven advances in SPR methodology, including improved surface chemistries, use of synthetic or defined oligosaccharides, and integration with orthogonal techniques such as mass spectrometry and NMR spectroscopy [15,16,17].

In this review, we provide a comprehensive overview of the use of SPR in studying GAG–protein interactions. We describe the fundamental principles of SPR, discuss key considerations in experimental design and data interpretation, and highlight representative applications in biomedical and pharmaceutical research. Finally, we outline emerging trends and future opportunities in leveraging SPR biosensors to deepen our understanding of GAG biology. By systematically linking SPR-derived kinetic parameters to biological function, disease relevance, and emerging therapeutic applications, this review aims to serve both as a practical guide for experimental design and as a conceptual framework for interpreting GAG–protein interactions in modern glycoscience research.

## 2. Principles of Surface Plasmon Resonance

SPR is an optical, label-free detection technique that enables real-time monitoring of biomolecular interactions by measuring changes in the refractive index near a metal–dielectric interface, typically gold [9,10,11,18]. Since its first application to biosensing in the 1980s, SPR has become a widely used tool in the study of protein–protein, protein–carbohydrate, and protein–nucleic acid interactions, owing to its sensitivity, quantitative capability, and adaptability to various biomolecular systems [19,20,21].

### 2.1. Principles of Surface Plasmon Resonance

SPR relies on the excitation of surface plasmons (coherent oscillations of conduction electrons at a metal–dielectric interface)at the interface between a metal (usually gold) and a dielectric (usually aqueous buffer) when polarized light is incident at a specific angle through a prism (Kretschmann configuration) [22,23]. When biomolecular binding occurs at the sensor surface, the local refractive index changes, which alters the angle or intensity of reflected light. This shift is recorded as a sensorgram, typically in resonance units (RUs), proportional to the mass of analyte bound at the surface [24].

The SPR sensorgram provides a real-time trace of the interaction, allowing for kinetic analysis by fitting the association and dissociation phases to mathematical models. The *ka*, *kd*, and *K_D_* can be extracted to characterize interaction affinity and kinetics [25].

### 2.2. Instrumentation and Formats

Modern SPR instruments are built on this optical principle but vary in configuration and throughput. Common systems include Biacore (Cytiva), Reichert, Nicoya, and Carterra platforms, each offering different sensitivity ranges, surface chemistries, and fluidic designs (see Table 1). Traditional flow-based systems are most common, but SPR imaging (SPRi) now enables parallel, high-throughput interaction screening across hundreds of immobilized ligands in a microarray format [26,27,28].

SPR experiments typically involve immobilizing one binding partner (ligand) on a sensor surface, while the second partner (analyte) is passed over in a controlled flow. Different immobilization strategies—such as amine coupling, thiol coupling, or affinity tag capture (e.g., biotin–streptavidin)—are selected based on the chemical properties of the ligand and the desired orientation or regeneration capability [29].

### 2.3. Advantages and Limitations in Glycosaminoglycan Studies

SPR is particularly advantageous for analyzing GAG–protein interactions because it enables label-free, real-time detection of binding events while preserving the native conformations of both ligand and analyte. The method is also well suited for characterizing transient or low-affinity interactions, offers high kinetic resolution for distinguishing fast and slow binders, and requires only small sample volumes—an important benefit when working with scarce or costly glycans and proteins.

However, challenges specific to GAG studies include non-specific electrostatic binding due to the high charge density of GAGs, heterogeneity in GAG structure, and potential mass transport limitations when working with large or multivalent proteins [30,31,32]. Carefully optimized surface chemistries, running buffers with defined ionic strength, and the use of well-characterized oligosaccharide ligands help mitigate these issues [12,33].

The combination of precise surface control, versatile ligand immobilization, and powerful kinetic analysis makes SPR a central method for dissecting the complex interactions between GAGs and their protein partners.

## 3. Experimental Design for GAG–Protein Studies by SPR

Effective analysis of glycosaminoglycan–protein interactions by SPR depends heavily on thoughtful experimental design. Due to the structural complexity and high charge density of GAGs, several parameters—including ligand immobilization, analyte purity, buffer composition, and surface regeneration—must be optimized to ensure reliable and interpretable results. This section outlines key considerations specific to GAG–protein systems.

### 3.1. Surface Immobilization Strategies

Immobilization of one binding partner (typically the GAG or protein) to the SPR sensor surface is a critical step that impacts orientation, accessibility, and activity. GAGs are often immobilized due to their structural flexibility and ease of chemical modification, although reversed configurations are also used depending on experimental goals [8,34].

Common immobilization methods for GAGs include the following:

**(1)**  **Amine coupling**: GAGs with free amino groups (e.g., via carbodiimide chemistry) are covalently attached to carboxymethyl dextran sensor chips (e.g., CM5). This method is robust but may reduce activity if key binding sites are involved [35].
**(2)**  **Biotin–streptavidin capture**: Biotinylated GAGs can be captured on streptavidin-coated chips, allowing for controlled orientation and regeneration. This is especially useful for sulfated oligosaccharides where preserving native binding epitopes is essential [36].
**(3)**  **Thiol coupling**: Less common, but useful when GAGs are derivatized with thiol groups to attach to maleimide-functionalized surfaces [37].

Alternatively, immobilizing the protein ligand—especially for recombinant proteins with affinity tags—may be preferred when the GAG analyte is available in multiple chain lengths or sulfation variants, allowing for comparative analysis in solution [38].

### 3.2. Selection and Preparation of GAGs and Proteins

GAG heterogeneity poses a challenge for reproducibility and mechanistic interpretation. Natural polysaccharide preparations (e.g., heparin, heparan sulfate) exhibit broad distributions of chain lengths and sulfation patterns. Therefore, the use of well-characterized defined oligosaccharides, chemoenzymatically synthesized structures, or synthetic mimetics is increasingly preferred [1,2,3,39].

Purified proteins must be carefully prepared to retain native conformation. When studying proteins with known GAG-binding domains (e.g., antithrombin, FGF2, CXCL12), inclusion of cofactors or divalent cations may be necessary. Potential glycosylation or post-translational modifications of the protein should also be considered, as they can influence binding affinity [33,40].

### 3.3. Buffer Composition and Ionic Conditions

SPR assays of GAG–protein interactions are highly sensitive to buffer composition because electrostatic forces dominate these binding events. Variations in ionic strength, pH, and divalent cation content can markedly influence both specific and non-specific binding responses [25]. Common buffer considerations include maintaining physiological salt concentrations (e.g., ~150 mM NaCl) to suppress non-specific electrostatic interactions, selecting an appropriate pH range (typically pH 6.5–7.5) based on the protein’s isoelectric point, and incorporating low concentrations of detergents (0.005–0.05% Tween-20) to reduce surface adsorption. In cases where protein activity or ligand recognition requires metal ions, cofactors such as Ca^2+^ or Mg^2+^ may be added (as is well-documented for selectins and integrins). In practice, buffer optimization is essential for maximizing signal-to-noise ratios and minimizing experimental artifacts.

### 3.4. Surface Regeneration

Following each SPR binding cycle, sensor surfaces must be regenerated to remove bound analyte and permit repeated measurements. For GAG-presenting surfaces, regeneration can be particularly challenging because these ligands are often labile and engage proteins through multivalent, high-avidity interactions. Common regeneration approaches include brief exposure to low-pH solutions (e.g., 10 mM glycine–HCl, pH 2.0–2.5) to disrupt electrostatic contacts, high-salt solutions (such as 2 M NaCl) to weaken charge-based interaction [41], and, in some cases, chaotropic agents (e.g., 10 mM NaOH or SDS), although these should be used cautiously to avoid damaging the immobilized GAG layer. In all cases, regeneration conditions must be determined empirically to ensure efficient removal of bound protein while preserving the integrity and functionality of the GAG ligand.

### 3.5. Controls and Validation

To ensure reliable interpretation of SPR data—particularly when analyzing complex GAG–protein binding profiles—appropriate experimental controls are essential. Reference surfaces (such as blank channels, unmodified matrices, or surfaces presenting irrelevant GAGs) are required to account for bulk refractive index effects and non-specific binding. Negative control proteins that lack GAG-binding motifs help establish baseline responses and verify the absence of unintended interactions. In addition, competition experiments using soluble GAGs or other inhibitors provide an important means of confirming binding specificity and validating the observed interaction mechanism.

SPR measurements can be corroborated with orthogonal techniques such as isothermal titration calorimetry (ITC), fluorescence anisotropy, or glycan microarrays for a more comprehensive view of binding behavior [12].

## 4. Data Analysis and Interpretation

SPR provides not only qualitative insights into biomolecular interactions but also detailed quantitative parameters. When applied to GAG–protein interactions, careful interpretation of sensorgrams is essential due to the complex, often multivalent nature of binding. This section outlines key data analysis strategies, including kinetic modeling, binding stoichiometry, and competition assays, highlighting considerations unique to GAG–protein systems.

### 4.1. Binding Kinetics and Affinity

The primary advantage of SPR is the ability to monitor real-time association and dissociation events, from which kinetic parameters are extracted. The binding response (measured in resonance units, RU) is plotted over time in a sensorgram that reflects the interaction profile.

In most cases, the data are analyzed using a 1:1 Langmuir binding model, which assumes reversible binding between a single ligand and analyte:

A + B ⇌ AB,(1)

From the *ka* and *kd* rate constants, the *K_D_* can be calculated as follows:

*K_D_* = *kd*/*ka*, (2)

However, GAG–protein interactions often deviate from ideal 1:1 behavior due to multivalency, heterogeneous binding sites, or mass transport limitations [10,25,42]. As such, more complex models—such as bivalent analyte models or heterogeneous ligand models—may be required to adequately fit the data [9]. In such cases, although the interaction can be fitted with a 1:1 Langmuir model, the resulting *kd* values are often clearly non-physical. For example, in the study of Listeria monocytogenes internalin B (InlB), the authors initially applied a 1:1 Langmuir fit and obtained very high apparent affinities (*K_D_* ≈ 3–6 nM). However, Scatchard analysis showed curvature, and the Hill coefficient was >1, which indicates weak positive cooperation, meaning that the interaction does not follow a simple 1:1 Langmuir model. Kinetic inspection further showed that *kd* depended on the contact time, and the dissociation phase changed with flow rate, consistent with mass-transfer coupling and surface rebinding. Accordingly, the authors adopted a two-site/heterogeneous ligand model for global fitting and emphasized that the derived *ka*, *kd*, and *K_D_* should be interpreted as apparent parameters.

For weak, fast-dissociating interactions (*K_D_* in the µM–mM range), steady-state analysis may be preferred over kinetic modeling. In this approach, RU values at equilibrium are plotted against analyte concentration and fitted to a binding isotherm to extract *K_D_* values [43].

### 4.2. Stoichiometry and Binding Models

GAG–protein interactions frequently involve multiple binding domains or clusters of positively charged residues on the protein surface. As a result, SPR sensorgrams may exhibit complex features such as non-linear association phases, biphasic dissociation behavior, or incomplete regeneration. These responses can reflect several underlying mechanisms, including heterogeneity within the immobilized GAG population (e.g., variable sulfation patterns), multivalent binding in which a single protein engages multiple GAG chains simultaneously, or protein conformational changes that occur upon ligand engagement [34]. Curve fitting using bivalent or heterogeneous models can help capture these dynamics. Global fitting—simultaneous fitting of multiple concentrations to a shared kinetic model—enhances the robustness and interpretability of kinetic parameters [44].

### 4.3. Thermodynamics and Competition Assays

Thermodynamic analysis can provide additional information about the driving forces of GAG–protein interactions. By performing SPR at multiple temperatures and applying the van’t Hoff equation, enthalpic (ΔH) and entropic (ΔS) contributions can be estimated:

ln *K_D_* = −RTΔH + RΔS (3)

This approach reveals whether binding is dominated by hydrogen bonding, ionic interactions, or hydrophobic effects—critical for rational drug design and structure–function analysis [45].

Competition assays are also widely used in SPR to assess binding specificity or screen potential inhibitors. In these assays, soluble GAGs or mimetics are pre-mixed with the analyte before injection. A reduction in RU signal indicates competitive inhibition and allows estimation of IC_50_ values. This method is particularly effective for comparing sulfation-dependent binding preferences across a panel of GAG structures [12].

### 4.4. Quality Control and Data Validation

SPR data require careful interpretation to avoid overfitting or misrepresentation of binding behavior. Key controls and validation steps include subtraction of blank or reference signals to account for bulk refractive index changes, assessment of repeatability across replicate injections or sensor surfaces, verification of surface stability before and after regeneration, and orthogonal validation using techniques such as isothermal titration calorimetry (ITC), ELISA, or NMR to confirm binding mode and stoichiometry [46]. Robust data analysis tools, including BIAevaluation (Cytiva), TraceDrawer (Ridgeview Instruments), and Scrubber (BioLogic Software), provide model fitting, residual analysis, and kinetic visualization to ensure reliable interpretation.

## 5. Applications of SPR in GAG–Protein Interaction Studies

SPR has been widely applied to elucidate the binding properties of glycosaminoglycans GAGs with various proteins implicated in coagulation, inflammation, infection, and tissue development (Table 2). This section highlights representative examples where SPR has significantly advanced our understanding of GAG–protein interactions in biological and therapeutic contexts.

### 5.1. Coagulation and Antithrombotic Mechanisms

One of the earliest and most extensively studied GAG–protein interactions involves heparin and antithrombin III (AT III), which plays a central role in the regulation of blood coagulation. In the anticoagulant cascade, heparin first binds to AT III through the AT III-binding pentasaccharide (ABD) to form a binary complex, which exerts anticoagulant function by directly inhibiting Xa. However, for IIa, heparin needs to contain a thrombin-binding domain (TBD) to interact with IIa and form a heparin–AT III–IIa ternary complex to exert anticoagulant function. SPR studies have clarified the ABD specificity required for AT III activation, revealing how sulfation patterns and conformational changes influence binding affinity [76,77].

In the study on the anticoagulant activity of HS, Hernaiz et al. proposed that the binding of HS to AT III is much weaker than that of heparin [78]. Notably, the binding affinity of HS to AT III is significantly enhanced after modification by 3-O-sulfotransferase-1. Study further focuses on the mechanism of formation of the final complex directly involved in anticoagulation (the ternary complex of heparin–AT III–IIa). Although the binding of heparin to IIa is through TBD, not all heparin chains with local ABD and TBD exhibit anticoagulant activity. They should be separated by a spacer with tunable length and rigidity, enabling rational and directional control over the anti-Xa/anti-IIa anticoagulant profile. Longer synthetic constructs further reveal a clear chain length threshold for thrombin inhibition (≥16-mer), with potency increasing sharply with chain extension (e.g., IC_50_ ≈ 130 µg/mL for a 16-mer versus ≈ 6.7 µg/mL for a 20-mer)

Similarly, SPR has been instrumental in evaluating interactions of heparin with thrombin, factor Xa, and heparin cofactor II, allowing detailed kinetic characterization that has informed the development of low molecular weight heparins and synthetic anticoagulants [4]. It also enables real-time monitoring of drug binding to heparin, critical for neutralization studies with protamine sulfate [79].

Zhao et al. proposed a new method to quantify the anticoagulant activity potency of heparin using SPR [80]. Various sources and molecular weights of heparin were used in the study, and the measurement results of SPR were not significantly different from those of traditional chromogenic assays in accuracy and stability. SPR has obvious advantages over traditional anticoagulant measurement due to its rapid analysis and no additional processing of samples. In addition, SPR could monitor the interaction between drugs and heparin in real time, which is particularly critical for studying the neutralization effect of protamine sulfate on heparin.

### 5.2. Inflammation and Immune Modulation

GAGs regulate inflammation by binding to chemokines, cytokines, selectins, and toll-like receptors. Chemokines regulate inflammation in vivo by forming chemotactic gradients and driving leukocyte adhesion and extravasation. HS/heparin mediates inflammation by controlling the speed of gradient formation and the extent of signal retention, by fixing chemokines on the surfaces of tissues/blood vessels. Tanino et al. found in an SPR study of different chemokines that different chemokines showed different patterns in *ka* and *kd*: on the heparin chip, KC showed faster, biphasic binding/dissociation than MIP-2 and CXCL8, which exchanged more slowly, suggesting that KC is more conducive to establishing effective gradients and generating stronger neutrophil recruitment in the lung and a high-diffusion, high-perfusion environment [81]. For MIP-2 and CXCL8, it is easier to form a more defined and stable gradient. In further studies, CXCL8 mutants did not show detectable heparin binding, which may explain why these mutants diffuse more readily and are more widely distributed in vivo. In addition to kinetics, HS-coated SPR studies of human CCL5/RANTES support GAG-driven cooperative binding through HS-induced oligomerization, and oligomerization-deficient mutants also exhibit reduced chemotaxis [82].

After chemokine-mediated local enrichment and gradient formation, inflammatory cells extravasate through the adhesion cascade, and capture and rolling mediated by P-, E-, and L-selectin determine whether leukocytes can achieve initial adhesion under shear flow. Wang et al. proposed that, in addition to the classical recognition of sialylated Lewis epitopes by selectins, highly sulfated GAGs can act as direct ligands for selectins and competitively block these interactions, thereby establishing a quantifiable link between the structural state of GAGs in the glycocalyx/ECM and adhesion events [83]. Their study showed a high-affinity, stable, direct interaction between P-selectin and heparin, characterized by relatively slow dissociation kinetics. Further analyses of glycan dependence revealed clear structure–activity features in selectin–GAG interactions: CS-E and oversulfated CS/DS chains bound L- and P-selectin at nanomolar affinity, and even tetrasaccharides with repeated CS-E retained detectable binding, suggesting the presence of a minimal, structurally defined recognition motif [84]. Consistent with these findings, in vivo and pharmacological studies have suggested that heparin’s anti-inflammatory activity can be partly attributed to interference with L-/P-selectin-mediated adhesion steps and is sensitive to specific structural states (e.g., glucosamine 6-O-sulfation). These provide a rationale for developing GAG derivatives that preserve selectin inhibition while reducing anticoagulant activity [85].

### 5.3. Pathogen–GAG Interactions

Many pathogens exploit host cell-surface GAGs for attachment and entry. SPR has enabled the characterization of viral envelope protein–heparan sulfate interactions, including those from HIV-1 (gp120), dengue virus (E protein), and SARS-CoV-2 (spike protein) [86,87]. The invasion of HIV-1 initiated with gp120 binding to CD4, and further conformational rearrangements. In addition to receptor engagement, gp120 also interacts with cell-surface heparan sulfate (HS), promoting virion enrichment at the membrane and influencing downstream entry steps. Vivès’ study showed that CD4-induced conformational changes in gp120 significantly enhance HS binding, and that the HS–binding interface is not limited to the V3 loop but also involves CD4-induced/coreceptor-related regions [88]. These defined heparin/HS oligosaccharides of specific degrees of polymerization can target this site at nanomolar concentrations, suggesting that HS acts at a relatively late stage of attachment. Similarly, Moulard et al. reported that polyanion/glycosaminoglycan (GAG) binding involves a more conserved coreceptor-binding surface and is more readily detected for ×4 or R5 × 4 gp120, indicating that GAG binding is associated with tropism-related charge distribution and epitope exposure [61]. Subsequent studies further delineated four HS/heparin-binding domains (V2, V3, the C-terminus, and the CD4-induced bridging sheet), highlighting a multisite, conformationally coupled gp120–HS interface that supports the development of HS/heparin mimetics as entry inhibitors [89].

In SARS-CoV-2 entry, host cell-surface HSPGs facilitate viral enrichment at the plasma membrane and thereby promote infection. Accordingly, Kwon et al. first applied a heparin chip SPR-based competitive binding strategy to screen highly sulfated polysaccharides as “glycan vaccines” capable of disrupting HS–spike complex formation [90]. Marine polysaccharides, with their distinctive scaffolds and bioactivities, represent a particularly rich source of sulfated glycans for this purpose. Notably, holothurian sulfated glycans can function as HS mimetics and interfere with SARS-CoV-2, and their activity is not dictated solely by charge density but depends more strongly on the backbone type, sulfation pattern, and chain length/multivalency [62]. Using *Pentacta pygmaea* fucosylated chondroitin sulfate (PpFucCS), Dwivedi et al. reported that both anticoagulant and anti–SARS-CoV-2 activities correlate with molecular weight, suggesting that anticoagulant liability may be reduced by tuning chain length while retaining antiviral potency [91]. They further showed that a low-anticoagulant sulfated fucan from *Thyonella gemmata* exhibits significant antiviral activity against WT and Delta, supporting marine sulfated glycans as a source of safer lead scaffold [92]. Given the rapid evolution of SARS-CoV-2, He et al. extended SPR-based screening to the Omicron XBB.1.5 variant [93]. Using heparin chips with WT and XBB.1.5 spike RBDs, they evaluated competitive inhibition by 10 marine sulfated glycans (including sulfated fucans, FucCS, and fucoidans) against RBD–heparin binding. These candidates showed strong inhibition for both WT and XBB.1.5, indicating that marine HS mimetics may provide cross-variant, broad-spectrum potential and highlighting the promise of non-classical GAG architectures such as FucCS (a CS backbone with fucose branches) as SARS-CoV-2 glycan-vaccine leads.

In the early stage of dengue virus entry, the interaction between the envelope protein E and cell-surface HS/HSPGs is thought to facilitate initial attachment and enrichment of virions on the plasma membrane. Accordingly, heparin chip-based SPR platforms are widely used to quantitatively characterize this binding and to screen inhibitors. Marks et al. first established a competitive format using a heparin chip and evaluated polyanionic/glycosaminoglycan derivatives to derive kinetic parameters, thereby defining structural requirements for E protein recognition of polyanionic ligands [94]. Subsequently, Vervaeke et al. focused on domain III (DIII) of E and showed that highly sulfated *E. coli* K5 derivatives bind DIII and inhibit viral attachment/entry at nanomolar concentrations [95]. More recently, Yang et al. analyzed the high-affinity interaction between DENV-2 E protein and heparin using solution competition SPR (*K_D_* ≈ 8.83 nM) and compared the inhibitory activities of multiple sulfated glycans [65]. Among the fucosylated chondroitin sulfates (FucCS), PpFucCS was particularly potent, showing >80% inhibition of E–heparin binding at 5 μg/mL and an IC_50_ of 126 ng/mL, substantially outperforming heparin (IC_50_ ≈ 5495 ng/mL), consistent with stronger functional competitive binding.

SPR has also revealed bacterial virulence factors that recognize host GAGs, such as *C. diff* toxins [75], Listeria internalins, and *Streptococcus pyogenes* adhesins, offering mechanistic insights for antimicrobial targeting [96]. Zhang et al. showed that heparin binds *Clostridioides difficile* toxins A and B with high affinity (toxin A, *K_D_* = 3.3 nM; toxin B, *K_D_* = 13.5 nM), and that binding depends on both chain length and sulfation. Moreover, heparin and non-anticoagulant heparin inhibited *C. difficile* toxin A-induced cell rounding, supporting the viewpoint that exogenous heparin could act as a decoy ligand to competitively block toxin activity. Similarly, Listeria monocytogenes internalin B (InlB) also exhibits high affinity for heparin: binding increases markedly when the chain length ≥ dp14, with affinities in the nanomolar range, consistent with a role for heparin/HS in potentiating InlB–Met signaling during invasion.

### 5.4. Growth Factors and Developmental Regulation

Several growth factors, such as fibroblast growth factors (FGFs), vascular endothelial growth factor (VEGF), and hepatocyte growth factor (HGF), require heparan sulfate for receptor binding and signal activation.

**Figure 3:**
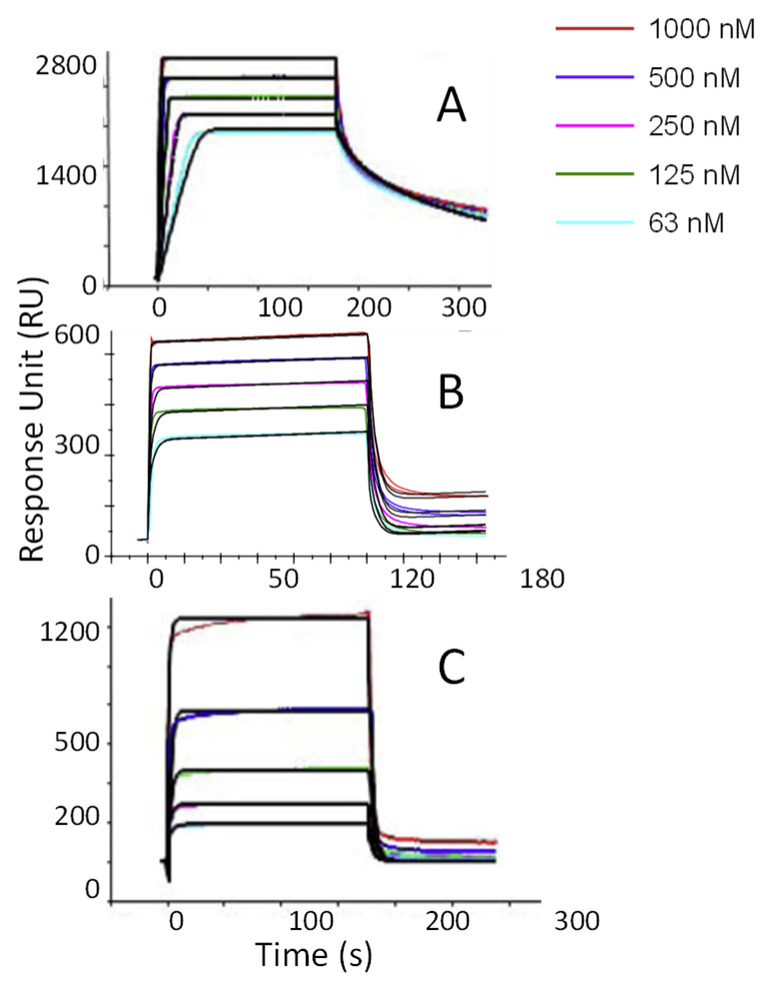
SPR sensorgrams of FGF2–GAG interactions. (**A**) FGF2–HS interaction; (**B**) FGF2–CSE interaction; (**C**) FGF2–KS interaction. Concentrations of FGF2 injected (from top to bottom) were as follows: 1000, 500, 250, 125, and 63 nM; the black curves are the fitting curves using model from BiaEvaluation Software. Data adapted from our previous studies [52,53].

FGFs are a family of signaling proteins that activate downstream phosphorylation cascades by inducing FGFR dimerization at the cell surface and are involved in diverse physiological processes, including development, homeostasis, tissue repair, and angiogenesis. Among them, FGF1 and FGF2 are important factors to promote mitosis and angiogenesis. HSPG on the cell surface has been found to play an important role in it signaling pathway. Guerrini et al. showed that tetrasaccharide can promotes FGF1 dimerization, and 6-O-sulfate plays an indispensable role in it, and the lack of 6-O-sulfate loses all activity [97]. Similarly, Delehedde et al., using different polymerized heparinsto study the minimum binding unit of FGF2, tetrasaccharide is also the smallest binding unit of FGF2 [97]. However, this study found that there was an obvious chain dependence in the binding between dp4-dp8, while dp10-dp14 was abnormal, which was mainly caused by the gradual reduction in *k_a_*. This may be because, since the conformational freedom of oligosaccharides ≥ dp10 is multiple, so the favorable conformation suitable for FGF2 binding is less likely to exist, resulting in the reduction in the proportion of productive collisions. In the subsequent study of phosphorylation biological activity, the longer chain still has higher activity, indicating that the decrease in affinity will not affect biological activity in some cases. Moreover, FGF2 not only interacts with HS/heparin but also exhibits substantial binding to CSE and KS (Figure 3).

The formation of an FGF–FGFR–HS ternary complex is a crucial step in its signaling pathway, and the assembly mode of its ternary complex becomes a key step. Ibrahimi’s research shows that the affinity between HSPG and FGFR on the cell surface is too low, so it is impossible to recruit FGF after the formation of a complex between HSPG and FGFR. Instead, HSPG should first interact with FGF or the FGF–FGFR complex [98]. Zhang F et al. further explored that the stability of the binary combination of FGF and HS was weaker than that of the complex of FGF–FGFR and HS [99]. The 6-O- sulfate of HS is not only important for the binding with FGF, but is also important for the binding with FGFR. However, not all binary complexes of FGF and FGFR have the same requirements for HS structure. FGF2–FGFR1 is more likely to bind to dp10 with a high proportion of 2SNS, while FGF2–FGFR2 is more likely to bind to dp10 with a high proportion of TriS. [100]. These insights have been essential in designing GAG-based scaffolds for tissue engineering and regenerative medicine.

### 5.5. Drug Discovery and High-Throughput Screening

SPR is a valuable platform for drug discovery targeting GAG–protein interactions, especially in high-throughput formats. Immobilized GAGs can be used to screen chemical libraries or natural product extracts for inhibitors that block pathogenic GAG–protein binding events [101].

SPRi and array-based systems allow parallel screening of diverse GAG structures, facilitating structure–activity relationship (SAR) analysis for potential therapeutics. For example, screening of heparin mimetics has led to the identification of small molecules that inhibit FGF or chemokine binding with sub-micromolar affinities [102].

SPR also enables competition assays to evaluate the potency of GAG analogs or neutralizing antibodies, helping prioritize candidates for preclinical development.

## 6. Challenges and Limitations

Despite its versatility and sensitivity, SPR faces several challenges when applied to GAG–protein interaction studies. These challenges stem from both the physicochemical complexity of GAGs and the technical constraints of SPR instrumentation. Understanding these limitations is essential to ensure accurate data interpretation and guide appropriate experimental design.

### 6.1. Immobilization Artifacts

SPR experiments typically require immobilization of one binding partner—most often the GAG—onto the sensor surface. However, this immobilization can introduce several artifacts, including alteration of the native GAG conformation, restricted ligand mobility, exposure of non-physiological binding sites, and the creation of heterogeneous surfaces [25]. Such effects can distort kinetic profiles or generate artificial binding events. Approaches including biotin–streptavidin capture, hydrazide coupling, or click chemistry can improve ligand orientation and surface stability, although these strategies may not fully reproduce the behavior of GAGs in solution [103].

### 6.2. Multivalency and Heterogeneity

GAG–protein interactions are frequently multivalent and predominantly governed by electrostatic forces, leading to complex binding behaviors. These include non-Langmuir kinetics, cooperative or avidity effects, and variable stoichiometry [12]. Additionally, GAGs like heparan sulfate exhibit extensive microheterogeneity in length and sulfation patterns, complicating data interpretation and reproducibility. Interactions may involve multiple low-affinity sites rather than a single high-affinity binding domain [104].

### 6.3. Mass Transport Limitations

When analyte concentrations are high or binding is very rapid, mass transport (i.e., diffusion to the sensor surface) may become rate-limiting. This produces curved sensorgrams that deviate from ideal kinetic fits and can lead to the underestimation of association rates (*ka*) [34]. Techniques such as lowering ligand density, increasing flow rate, or performing global fitting with transport-corrected models can help mitigate these effects but add complexity to data analysis [32].

### 6.4. Surface Regeneration Issues

Efficient regeneration of the sensor surface is essential for reproducible SPR measurements. However, strong or multivalent GAG–protein interactions can resist regeneration or denature ligands, limiting reuse of the sensor chip. Harsh regeneration conditions (e.g., high salt, low pH, detergents) may degrade immobilized GAGs or alter their activity [105].

### 6.5. Limited Structural Resolution

Although SPR offers detailed kinetic and affinity information, it does not directly provide insights into atomic-level binding sites, conformational changes, or allosteric mechanisms. Therefore, SPR is best complemented by structural techniques such as NMR spectroscopy, X-ray crystallography, cryo-EM, or molecular dynamics simulations for a complete understanding of GAG–protein complexes [3].

### 6.6. Non-Specific and Electrostatic Binding

Owing to their highly anionic character, GAGs frequently exhibit non-specific interactions with positively charged proteins, especially under low ionic strength conditions. Such interactions can result in elevated baseline responses, non-saturable binding curves, and inaccurate estimates of the *K_D_* [32]. Buffer optimization (e.g., increasing salt concentration) and the use of reference surfaces or control GAGs are essential to minimize these effects.

### 6.7. Biological Relevance of Synthetic GAGs

While synthetic oligosaccharides offer greater structural control, they may not accurately reflect native GAG conformations, presentation, or post-translational modifications. As a result, SPR data from synthetic analogs may not fully capture in vivo binding behavior, requiring cautious extrapolation [39].

## 7. Future Perspectives and Emerging Technologies

As the complexity and significance of GAG–protein interactions become increasingly recognized, the demand for more advanced, accurate, and high-throughput analytical tools continues to grow. SPR, already a cornerstone in biomolecular interaction studies, is evolving with new technologies that promise to overcome existing limitations and expand its utility.

### 7.1. SPR Imaging and Multiplexing Platforms

Recent advances in SPRi enable the real-time monitoring of hundreds of GAG–protein interactions simultaneously across arrayed surfaces. This high-throughput format facilitates rapid screening of diverse GAG structures or protein variants, systematic mapping of structure–function relationships, and comparative affinity profiling, which is particularly valuable for drug discovery and development [106]. Integration with microarray printing and robotic spotting systems allows miniaturized assays with minimal sample consumption, ideal for synthetic GAG libraries or clinical glycomics.

### 7.2. Microfluidic SPR and Lab-on-a-Chip Devices

The integration of SPR sensors with microfluidic platforms further enhances sensitivity, reproducibility, and multiplexing capabilities. These lab-on-a-chip systems provide automated buffer and sample handling, parallel channel architectures for simultaneous control and test surfaces, and precise flow regulation to reduce mass transport limitations. Microfluidic SPR also holds promise for point-of-care diagnostics, where rapid GAG-binding protein detection (e.g., viral spike proteins) could inform clinical decision-making [107].

### 7.3. Integration with Structural and Computational Methods

SPR data are increasingly complemented by techniques such as NMR, X-ray crystallography, cryo-EM, and molecular modeling to provide atomistic insights into GAG–protein interfaces. This integrative approach facilitates the identification of binding hotspots, the interpretation of complex kinetic behaviors, and the rational design of structure-based ligands [3]. Molecular dynamics (MD) simulations, particularly those incorporating sulfation and charge dynamics, provide complementary insights into SPR-derived interaction parameters and GAG flexibility [40].

### 7.4. Artificial Intelligence and Data-Driven Modeling

With the increasing scale and complexity of SPR datasets, machine learning (ML) and artificial intelligence (AI) are being applied to predict GAG-binding sites from sequence or structural information, model kinetic binding behaviors across diverse GAG variants, and analyze non-canonical or complex sensorgram profiles [108]. AI-assisted platforms can also suggest optimal experimental conditions, minimize false positives from non-specific binding, and assist in hit prioritization in drug discovery workflows.

### 7.5. Synthetic GAG Libraries and Chemical Biology Tools

Advances in chemoenzymatic synthesis and click chemistry now permit the generation of GAGs with precise chain lengths, sulfation patterns, and backbone modifications. These well-defined glycans facilitate high-fidelity SPR analyses, enable detailed characterization of protein specificity, particularly for chemokines and growth factors, and support the development of synthetic GAG analogs with therapeutic potential [109]. Photoaffinity probes and biotinylated analogs further expand SPR capabilities, allowing capture of transient or weak binders in complex biological samples.

### 7.6. Clinical and Diagnostic Applications

SPR-based biosensors are increasingly being investigated for clinical applications, such as detecting heparin contamination, monitoring anti-GAG antibody levels in autoimmune or cancer patients, and enabling rapid viral detection through GAG–spike protein interactions, as exemplified by SARS-CoV-2 [110]. The development of portable, robust SPR platforms with streamlined workflows holds promise for broader implementation in hospitals, research clinics, and pharmaceutical quality control laboratories.

### 7.7. Toward Systems Glycobiology

Ultimately, incorporating SPR into systems-level investigations of the glycocalyx—the complex extracellular matrix of GAGs and proteins—necessitates real-time, multivalent binding models, dynamic simulations of ligand competition and presentation, and integration with proteomic, glycomic, and single-cell analytical approaches [108]. Such holistic approaches will be essential to understand how GAG–protein networks regulate signaling, development, infection, and disease.

## 8. Conclusions

The study of GAG–protein interactions is critical for advancing our understanding of numerous biological processes, from cell signaling and development to immunity and disease pathogenesis. SPR has proven to be a valuable tool in this domain, offering real-time, label-free analysis of binding kinetics and affinity under physiological and pathological relevant conditions.

This review has highlighted how SPR facilitates detailed characterization of GAG–protein interactions through various immobilization strategies, kinetic modeling techniques, and increasingly sophisticated surface chemistries. Despite challenges such as GAG heterogeneity, multivalency, and electrostatic complexity, SPR continues to provide high-quality insights when combined with appropriate experimental design and complementary analytical techniques.

Technological innovations—including SPR imaging, microfluidic integration, synthetic GAG libraries, and machine learning algorithms—are poised to further enhance the power and applicability of SPR in glycoscience. As these tools evolve, they will play a central role in bridging fundamental research with translational goals in diagnostics, drug discovery, and personalized medicine.

In conclusion, SPR is not only a foundational method for exploring GAG–protein interactions but also a rapidly advancing biosensor platform that is increasingly aligned with the multidisciplinary demands of modern glycomics and systems biology.

## Figures and Tables

**Table 1:** Comparison of representative SPR/LSPR platforms and instrument models.

Manufacturer	Model	Detection Mode	Throughput	Chip Type	Features
Cytiva (Biacore)	T200	single-spot	4 flow cells	Series S chips: CM5, SA, NTA	Classic Biacore workflows;
	S200		4 flow cells		Better resolves fast kinetics/low signals: low noise, sampling for rapid off-rates
	X100		2 flow cells		Routine *K_D_* and reproducibility testing; lower cost
	1 series		6 flow cells		More flow paths; ideal for low response, difficult samples, unstable binders
	8 series		8 channels × 2 flow cells		Ultra-high throughput, robust analytics; handles complex matrices; fully automated
AMETEK Reichert	2SPR	single-spot	2 channels	Streptavidin-dextran surface, gold surface	Easy operation, crude/complex matrices
	3SPR		3 channels		Parallel assays for crude samples
	4SPR		4 channels		More channel options for crude samples
Nicoya	OpenSPR	LSPR	2 channels	Carboxyl, NTA, Streptavidin, Biotin, Protein A	Benchtop, low maintenance; diverse surfaces/sensors, including high-sensitivity sensors
	OpenSPR-XT		2 channels, 2 × 96-well plates		2 × 96-well plates enable automated MCK/SCK; sample cooling 22→−4 °C.
	Alto	Digital SPR	16 channels		Ultra-low sample, highly automated; disposable cartridges; digital microfluidics
Carterra	LSA	High-throughput SPR (array-based/imaging workflow)	384 spots	CMD/HC (and related derivatives)	High-throughput antibody/protein profiling for early screening; 96-channel printhead or single large flow cell
	LSA XT		96-channel printhead, 384 spots		HT-SPR for drug discovery, higher sensitivity and quality
	Ultra		192 spots		Enhanced optics, thermal control, and microfluidics

**Table 2:** Representative GAG–protein interactions studied by SPR.

Protein Family	Protein Target	GAG Type	*K_D_* Range	Biological Roles	Reference
Serpins	Antithrombin	Heparin	3.6 nM	Anticoagulation	Zhang et al. 2022 [47]
	Heparin cofactor II	Heparin	223 nM	Anticoagulation	Zhang et al. 2004 [48]
Amyloid Proteins	Tau K18	Heparin	200 nM	Tau aggregation	Zhao et al. 2017 [49]
	Tau K19		~70 µM		
	Aβ40	Heparin	15 nM	Amyloid aggregation	Wang et al. 2021 [50]
Growth Factor	FGF-2	Heparin	1.2 nM	Angiogenesis, cell proliferation	Zhang et al. 2019 [51]
		HS	7.2 nM		Weyers et al. 2013 [52]
		CSE	1.63 µM	Angiogenesis, neural development	Cai et al. 2012 [53]
		KS	970 nM	Corneal epithelial repair and wound healing	Weyers et al. 2013 [52]
	FGF1	Heparin	22 nM	Angiogenesis, wound healing	Zhang et al. 2014 [54]
		HS	490 nM		Weyers et al. 2013 [52]
		CSE	4.12 µM	Angiogenesis, neural development	Cai et al. 2012 [53]
	FGF7 (KGF)	Heparin	4.9 nM	Epithelial wound healing	Zhang et al. 2019 [51]
	FGF10	Heparin	1.3 nM	Lung development, epithelial branching	Zhang et al. 2019 [51]
	HGF	Heparin	0.14 nM	Angiogenesis and tissue repair	Zhang et al. 2019 [51]
	TGF-β1	Heparin	59 nM	Latent complex storage and activation	Zhang et al. 2019 [51]
	VEGF165	Heparin	80 nM	Angiogenesis, GAG required for receptor activation	Zhao et al. 2012 [55]
Cytokine/Chemokines	PF4	Heparin	1.2 nM	HIT antibody complex formation	Zhang et al. 2022 [47]
	IL-7 (human)	Heparin	440 nM	Signal transduction	Zhang et al. 2012 [56]
	IL-8 (CXCL8)	Heparin	8.3 μM	Neutrophil recruitment	Gerlza et al. 2014 [57]
		HS	11.5 μM		
	IP-10 (CXCL10)	Heparin	1.03 nM	Th1 lymphocyte recruitment, inhibits angiogenesis	Dillemans et al. 2024 [58]
		HS	6.73 nM		
		CS A	33.4 nM		
	CXCL11	Heparin	2.3 nM	Endothelial/ECM immobilization and gradient formation for CXCR3^+^ trafficking	Dyer et al. 2016 [59]
		HS	6.0 nM		
		CS A	45 nM	Matrix retention that tunes local bioavailability of CXCL11	
	SDF-1α (CXCL12)	Heparin	4.5 nM	Chemokine gradient formation, stem cell homing	Dyer et al. 2016 [59]
		HS	61 nM		
	MCP-1 (CCL2)	Heparin	44 nM	Monocyte recruitment, controls systemic effects	Salanga et al. 2014 [60]
		HS	70 nM		
	RANTES (CCL5)	Heparin	3.7 nM	T cell recruitment, HIV coreceptor blocking	Dyer et al. 2016 [59]
		HS	4.5 nM		
		CS A	51 nM		
	MCP-3 (CCL7)	Heparin	100 nM	Endothelial/ECM immobilization and gradient formation for leukocyte trafficking.	Salanga et al. 2014 [60]
		HS	120 nM		
Viral Protein	gp120 (HIV-1)	Heparin	200 nM	Viral adhesion and entry	Moulard et al. 2000 [61]
	SARS-CoV-2 Spike	Heparin	94 nM	Viral entry enhancement	Dwivedi et al. 2021 [62]
	Hepatitis C envelope protein	Heparin	5.2 nM	Viral adhesion and entry	Barth et al. 2003 [63]
	MERS RBD	Heparin	29.4 nM	Viral entry enhancement	Yang et al. 2024 [64]
	Dengue virus envelope protein type 2	Heparin	8.83 nM	Viral entry and immune recognition	Yang et al. 2024 [65]
	Monkeypox A29 and A35	Heparin	~200 nM	Viral entry and immune recognition	He et al. 2023 [66]
Bacterial Adhesin	*N. meningitidis* NHBA	Heparin	52 nM	Vascular endothelium adhesion	Mubaiwa et al. 2018 [67]
		HS	1362 nM	Lower affinity, tissue specificity	
		CS	5.2 nM	tissue specificity	
	*Mycoplasma pneumoniae* P30 and P1-C	Heparin	~10–100 μM	Adhesion to host cells, motility, and virulence	Yang et al. 2024 [68]
Neuronal Guidance	Semaphorin 3A	Heparin	1.6 nM	Repulsive guidance,	Pérez et al. 2021 [69]
	Slit2	HS	330 nM	Axon guidance	Zhang et al. 2004 [70]
	Robo1	Heparin	~650 nM	Axon guidance	Zhang et al. 2013 [71]
	Noggin	HS	72.3 nM	BMP antagonist, modulates gradient	Heide et al. 2022 [72]
	Tie1	HS	3.14 nM	Regulation of angiogenesis/ang tie signaling	Griffin et al. 2020 [73]
		CSE	14.7 nM		Griffin et al. 2020 [73]
	Sonic Hedgehog (Shh)	Heparin	67.1 nM	Tissue patterning	Zhang et al. 2007 [74]
		HS	31.6 μM		
Hormone/toxin	*C. diff* toxin A	heparin	~3 nM	Disruption of the colonic epithelium	Zhang et al. 2025 [75]
	*C. diff* toxin B	heparin	~11 nM		

## Data Availability

Data will be available on request.
